# Neovascular glaucoma: a retrospective review from a tertiary center in China

**DOI:** 10.1186/s12886-016-0190-8

**Published:** 2016-01-27

**Authors:** Na Liao, Chaohong Li, Huilv Jiang, Aiwu Fang, Shengjie Zhou, Qinmei Wang

**Affiliations:** School of Optometry and Ophthalmology and Eye Hospital, Wenzhou Medical University, Wenzhou, Zhejiang China

**Keywords:** Neovascular glaucoma, Etiology, Glaucoma drainage device

## Abstract

**Background:**

The purpose of this study is to report the prevalence, etiology, treatment and outcomes of neovascular glaucoma (NVG) in a tertiary care ophthalmic center in China.

**Methods:**

Medical records of patients diagnosed as NVG at the Wenzhou Medical University between 2003 and 2014 were reviewed. Success was defined as IOP between 6 and 21 mmHg without topical or systemic glaucoma medications with retention of presenting visual acuity (VA).

**Results:**

NVG was diagnosed in 483 of 8306 (5.8 %) of all glaucoma patients. Etiology is reported for all 310 eyes of 284 patients managed in the department. Interventions depended on insurance as well as personal finances; outcomes are reported for the 149 eyes of 138 patients with complete data that met follow up requirements. Diabetic retinopathy (DR,39.7 %) was the major cause of NVG. Kaplan Meier survival analysis showed a success rate of 84.8 % at 1 year, 47.5 % at 3 years and 21.9 % at 5 years. Major interventions included glaucoma drainage device (GDD) in 103 eyes and trans-scleral cyclophotocoagulation (TSCPC) in 22 eyes. Complications were more common in the GDD group.

**Conclusions:**

NVG comprised 5.8 % of glaucoma patients seen in a tertiary Chinese hospital. DR was identified as the commonest cause and probably reflects the increasing prevalence of diabetes in China. Surgical interventions were partly determined by insurance status and personal finances. GDD was the commonest surgical intervention used and also had the most complications.

## Background

Neovascular glaucoma (NVG) is a secondary, refractory condition that accounts for 0.7–5.1 % of glaucoma in an Asian population [1, 2]. The condition is secondary to obstruction of the trabecular meshwork by neo-vascular membrane that develops in response to retinal ischemia [3, 4]. Current surgical options for intraocular pressure (IOP) control in NVG comprise augmented trabeculectomy, glaucoma drainage devices (GDD) and cyclophotocoagulation (CPC) while prognostic factors include young age, previous vitrectomy and post-surgery complications [4–6]. Recent prospective data on the management of refractory glaucoma from China reported GDD as safe and effective for some of the conditions but with a relatively poor outcome in NVG [7]. Late presentation and loss of follow up are additional challenges for the management of NVG in China [8].

There is paucity of data from China relating the causes and management of NVG. The objective of this study is to report the causes, management and outcomes of NVG in a tertiary facility in China.

## Methods

The study was approved by the ethics committee of the Wenzhou medical university. As this was a retrospective study with de-identified data informed consent was not required. The records of all patients diagnosed as NVG between June 2003 and March 2014 at the eye hospital of Wenzhou Medical University, Wenzhou, China were reviewed. The diagnosis of NVG was based on an IOP > 21 mmHg on applanation tonometry associated with neovascularization of the iris and/or angle of the anterior chamber detected by slit lamp bio-microscopy and gonioscopy [9]. All patients with NVG treated in our hospital were investigated to determine the cause. Other data extracted from the records included age, gender, affected eye, visual acuity(VA), lens status, IOP, number of glaucoma medications used at presentation and at last visit, types of intervention, history of previous intraocular surgery, use of anti-VEGF agents and post operative complications. Etiology of NVG was reported for all patients managed in the hospital. Patients with a follow up of less than 6 months were excluded, as were those without IOP and VA data at the final visit.

All patients received treatment for the underlying cause as well as medical treatment for control of IOP. Surgery was undertaken if further IOP lowering was deemed necessary either for retention of vision or for comfort. The overall management however depended not only on surgeon preference but also on insurance cover as well as capacity to pay. While 95 % of our patients had medical insurance, reimbursement is not uniform and payment for prosthesis like GDD is usual.

Surgery if indicated was undertaken by one of 5 surgeons. For patients with useful vision, most surgeons preferred GDD as the first surgical option. Trabeculectomy was offered for such patients if economic factors precluded the use of a GDD, while trans-scleral cyclophotocoagulation (TSCPC) was generally offered those without useful vision. Endoscopic cyclophotocoagulation (Endo-CPC) was introduced in our hospital in 2006 and was combined with cataract surgery/vitrectomy or used in isolation for refractory NVG in some pseduophakic eyes. Anti-vascular endothelial growth factor (anti-VEGF) became available in the clinic from 2008 and was used on a case-by-case basis determined primarily by insurance cover and affordability.

All GDD surgery was performed using Ahmed implants (New World Medical, Inc., Rancho Cucamonga, CA, USA). The surgical technique used for GDD and trabeculectomy were similar to that described in the literature [10, 11]. All trabeculectomies were performed with adjunctive mitomycin C, but the decision to use mitomycin with GDD was made by the individual surgeon. TSCPC was performed using the G probe with the diode laser (Iridex Corporation, Mountain View, CA, USA); Endo-CPC; was undertaken with the Endo-OPTIKS machine (Endo-OPTIKS, Little Silver, NJ, USA). The technique of TSCPC and Endo-CPC used was similar to that described [12, 13]. For TSCPC, three to four quadrants were treated with the G probe with about 20 spots avoiding the 3 and 9 o’clock positions with parameters adjusted to avoid a ‘pop’. For Endo-CPC about 200 degrees of ciliary processes were coagulated.

Success was defined as an IOP between 6 and 21 mmHg without topical or systemic glaucoma medications with retention of presenting VA; this visual criteria was applied to patients with a VA of light perception or better [5]. Snellen’s VA was converted to the logarithm of the minimal angle of resolution (LogMAR) for analysis. An improvement or decrease in visual acuity was defined as a change of two or more lines on the LogMAR scale. LogMAR values for low vision were defined as follows: counting fingers (CF) =2.3; hand motions (HM) =2.6; light perception (LP) =2.9; and no light perception (NLP) =4 [14]. For those with low vision, a difference of one low vision category post-surgery was considered as change. Patients with incomplete data (lack of IOP and VA) and follow up less than 6 months were excluded from the analysis of outcomes.

### Statistical analysis

All statistical analyses were performed using SPSS software version 15.0 (SPSS, Inc., Chicago, IL) and *p* < 0.05 was considered significant. Normality of variables was ascertained using Kolmogorov-Smirnov tests. Continuous normally distributed data were presented as mean ± standard deviation; proportions (%) were used to describe categorical variables while non-normal distribution variables were presented as median (interquartile range). The paired student *t*-test and Wilcoxon signed-rank test was performed to compare the IOP and mean number of anti-glaucoma medications at presentation and at last visit respectively. Kaplan Meir survival analysis was used to report success rates. Cox’s proportional hazards regression model [odds ratio (OR) with 95 % confidence interval (CI)] was used to report the association of potential prognostic factors: age, binocular involvement, lens status, previous intraocular surgery (vitrectomy or cataract surgery), systemic diseases, use anti-VEGF for underlying cause, history of PRP and influence of postoperative complications.

## Results

NVG was diagnosed in a total of 483 (5.8 %) of the 8306 glaucoma patients seen during the study period. 199 patients elected to seek care elsewhere while 284 were investigated and managed at the Wenzhou medical university eye department. Etiology of NVG was established and reported for these 284 patients. After excluding 14 patients lacking IOP or VA data at their last visit and 132 patients with less than 6 months follow up, 149 eyes of 138 patients (85 males and 53 females) were available for reporting interventions and outcomes.

Patient demographics are shown in Table 1. All patients were Han-Chinese. The mean age was 64.2 ± 14.0 years (range, 10–94 years) and the median follow-up 18.5 months (range, 6.07–103.8 months). There were 105 phakic (70.5 %), 11 aphakic (7.4 %) and 33 pseudophakic (22.1 %) eyes. The causes of NVG are listed in Table 2. Diabetic retinopathy (123/310 eyes, 39.7 %) was the commonest followed by CRVO (66/310 eyes, 21.3 %). The primary cause could not be determined in 59 eyes (19.0 %). 26 patients had bilateral NVG; 15 of these patients presented with bilateral NVG while 11 were diagnosed in the fellow eye after 1 to 16 months. 20/26 (76.2 %) cases with bilateral NVG were caused by DR, two were due to bilateral CRVO, one had bilateral uveitis, one was ascribed to bilateral BRVO, one was caused by the ocular ischemia syndrome while the cause in one bilateral case remained unknown.

**Table 1:** Demographic Data

Clinical characteristics of eyes with neovascular glaucoma (*N* = 149)	
Age in years (range)	64.2 ± 14.0 (10–94)
Eyes (%)	
Right	76 (51.0 %)
Left	73 (49.0 %)
Gender (%)	
Male	85 (61.6 %)
Female	53 (38.4 %)
Lens (%)	
Phakic	105 (70.5 %)
Aphakic	11 (7.4 %)
Pseudophakic	33 (22.1 %)
Presenting Vision	
Improvement VA	21 (22.6 %)
Unchanged^a^	34 (36.6 %)
Decrease VA	38 (40.9 %)
Decrease to NLP	16 (17.2 %)
Previous VA = 1	2
Previous VA = CF	1
Previous VA = HM	6
Previous VA = LP	7
IOP at presentation^b^	43.5 ± 10.9
IOP at final follow-up^b^	19.0 ± 11.4
Glaucoma medications	
Median at presentation (IQR)	2 (3)
Median at final follow up (IQR)	0 (1.5)
Median interval of follow up in months (IQR)	18.5 (17.6)
History of intraocular surgery	54 (36.2 %)
Complications	38 (25.5 %)
Enucleation (%)	5 (3.4 %)
Hypotony (%)	8 (5.4 %)

*IOP* intraocular pressure, *VA* visual acuity, *IQR* interquartile range

^a^56 eyes with NLP at presentation were excluded

^b^5 enucleation and 2 phthisis bulbi excluded

**Table 2:** Etiological Factors Associated with NVG

Causes of neovascular glaucoma	Eyes (%)
Retinal ischemic disease	
DR	123 (39.7)
CRVO	66 (21.3)
BRVO	5 (1.6)
CRAO	3 (1.0)
RD	17 (5.5)
Others^a^	9 (2.9)
Ocular ischemia syndrome	7 (2.3)
Uveitis	6 (1.9)
Trauma	13 (4.2)
Surgical causes	3 (1.0)
Radiation	1 (0.3)
Unknown	59 (19.0)

*DR* diabetic retinopathy, *CRVO* central retinal vein occlusion, *CRAO* central retinal artery occlusion, *BRVO* branch retinal vein occlusion, *RD* retinal detachment

^a^Including Coat’s exudative retinopathy (4), Ischemic optic neuropathy (2), Hypertensive retinopathy (1) and Persistent Hyperplastic Primary Vitreous (1) and Eales’ disease (1)

In eyes with presenting VA of LP or better (93 eyes of 86 patients), VA was retained in 34 eyes (36.6 %), decreased in 38 eyes (40.9 %) and improved in 21 (22.6 %) eyes. Deterioration of vision to NLP occurred in 16/93 (17.2 %) eyes. Two of these 16 eyes had useful VA (LogMAR = 1) prior to surgery. One case was caused by severe DR while the other was caused by BRVO. Both were managed with a GDD; one case developed retinal detachment.

Of the 149 eyes, two developed phthisis bulbi and 5 required enucleation. Excluding these 7 eyes, IOP decreased from a mean (± SD) of 43.5 ± 10.9 to 19.0 ± 11.4 mmHg (*p* = 0.000). The number of glaucoma medications was reduced from 2 (interquartile range =3) to 0 (interquartile range = 1.5) after interventions (*p* = 0.000)

**Fig. 1:**
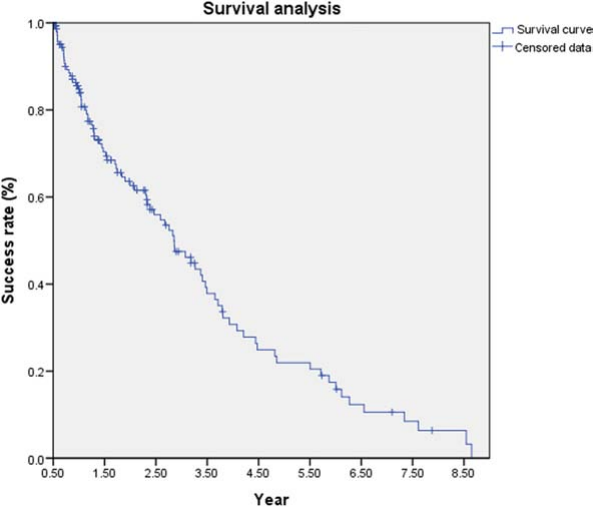
Kaplan-Meier survival curve of treatment outcomes in all 138 NVG patients (149 eyes). Censored data relates to the ones who had not observed failure at that time (including missing data)

Kaplan-Meier survival analysis is shown in Fig. 1. Success was achieved in 84.8 % at 1 year, 47.5 % at 3 years and 21.9 % at 5 years. Six eyes that required combinations of GDD, TSCPC or trabeculectomy were excluded from this analysis (3 eyes that underwent TSCPC followed by GDD or Trabeculectomy and 3 that required TSCPC following GDD). Of the remaining 143 eyes, 103 eyes underwent GDD, 22 eyes were treated with TSCPC, 7 eyes had trabeculectomy, 3 eyes underwent Endo-CPC and 8 eyes underwent combined surgery. Table 3 details the treatment modalities and their outcomes. Age, lens status, the use of anti-VEGF, postoperative complications, PRP, prior-intraocular surgery did not have a statistically significant influence on outcome (Table 4).

**Table 3:** Treatment and results (*N* = 143 eyes)^a^

Treatments[N(%)]	No. cases	Complete success rate	Decreased VA	The percentage of IOP reduction
TB	7 (4.9 %)	4 (57.1 %)	2 (28.6 %)	70.0 %
GDD	103 (72.0 %)	46 (32.2 %)	35 (34.0 %)	54.8 %^b^
TSCPC	22 (15.4 %)	7 (31.8 %)	4 (18.2 %)	66.8 %^c^
Endo-CPC	3 (2.1 %)	0	3 (100 %)	17.2 %^d^
Combined surgery	8 (5.6 %)	4 (50.0 %)	1 (12.5 %)	61.1 %
Vitrectomy & GDD	3	1	0	48.2 %
Cataract & GDD	3	3	0	73.6 %
Cataract & Endo-CPC	2	0	1	60.5 %

*TB* trabeculectomy, *GDD* glaucoma drainage devices, *TSCPC* trans sclera cyclophotocoagulation, *Endo-CPC* endoscopic cyclophotocoagulation

^a^6 eyes treated with multiple operations excluded

^b^GDD group excludes 1 enucleated eye

^c^TSCPC group excludes 3 enucleated eyes and 1 with phthisis bulbi

^d^Endo-CPC group excludes 1 eye with phthisis

**Table 4:** Prognostic factors

Parameters	*P*	Odds ratio	95 % confidence interval
Age	0.85	1.07	0.53–2.16
Binocular	0.34	1.36	0.72–2.56
Lens status	0.99	1.00	0.72–1.39
Prior surgery	0.78	1.10	0.59–2.02
Hypertension	0.34	1.28	0.77–2.11
Diabetes	0.18	0.69	0.40–1.19
Anti-VEGF	0.32	1.79	0.58–5.56
Complications	0.30	0.78	0.48–1.25
PRP	0.43	1.22	0.74–2.01

Postoperative complications are summarized in Table 5. Shallow anterior chamber was the commonest complication (12/143, 8.4 %) followed by hypotony (8/143, 5.6 %). Tube-related complications included occlusion/displacement of the tube and occurred in 2 eyes. Most complications occurred in the GDD group.

**Table 5:** Postoperative complications (*N* = 143 eyes)^a^

Complications	Number (%)	Group with highest incidence
Hyphema	4 (2.8)	GDD (4)
Shallow anterior chamber	12 (8.4)	GDD (10)
Choroidal detachment	2 (1.4)	GDD (2)
Ciliary body detachment	4 (2.8)	GDD (4)
Tube related complications^b^	2 (1.9)	-
Bleb-related complications^c^	3 (2.6)	GDD (3)
Hypotony	8 (5.6)	GDD (4)
Endophthalmitis	0	-

*GDD* glaucoma drainage devices

^a^6 eyes treated with multiple operations were excluded

^b^103 eyes treated with GDD were analyzed

^c^22 eyes treated with TSCPC, 3 with Endo-CPC and 2 with cataract & Endo-CPC were excluded

## Discussion

A survey by the Beijing union medical college hospital and WHO in 1996 reported that the prevalence of secondary glaucoma in the population over the age of 50 years was 0.12 % and accounted for 5.9 % of all glaucoma [15]. NVG per se was not reported, but as it is a secondary glaucoma, we can infer that the proportion of NVG in that survey was lower than 5.9 %. NVG was diagnosed in 5.8 % of 8306 glaucoma patients seen in our tertiary hospital. This is similar to the 6.7 % of 1232 Chinese subjects diagnosed as glaucoma in a population based survey in Singapore [16]. Although it cannot really be compared to population-based data, Our data provides the prevalence of NVG in a tertiary eye hospital in China.

DR (39.7 %) followed by CRVO (21.3 %) was the major cause of NVG in this series. While the etiology could only be determined for the 284 patients who elected to continue their care in our center, the pattern is consistent with reports from other countries [17–19]. A recent hospital based report from China provides conflicting results: CRVO (39.2 %) was reported as the commonest cause in 120 NVG eyes [20]. Diabetes, a major cause of NVG has increased in China over the years and has a current reported prevalence of 23 %, affecting an estimated 92.4 million adults in the mainland [21]. Our data seems to suggest a change in etiology of NVG that is likely linked to the increasing prevalence of diabetes in China. Lack of data and the retrospective nature of the study contributed to our inability to identify the cause in 19 % of cases.

VA results were sobering. Acuity was poor to start with and could only be preserved or improved in 59 % of cases while 41 % worsened, with 16 eyes deteriorating to no light perception. Improvement in vision occurred in 21/93 eyes (90 % due to resolution of corneal edema, hyphema and/or vitreous hemorrhage). GDD was the commonest surgical intervention used and also had the most complications. Interventions in our hospital depend on surgeon preference and experience as well as cover by medical insurance. GDD (103/143, 70.2 %) was the first option for controlling IOP in NVG patients with potential for vision. TSCPC was the second choice (22/143, 15.4 %) and tended to be used in those with a poorer prognosis.

Kaplan-Meier survival analysis showed a 84.8 probability of success at 1 year, 62.6 % at 2 years and 21.9 % at 5 years. Nakatake, etc. found that success rate of primary trabeculectomy (with or without anti-VEGF) prior to surgery was 70.9 % after 1 year, decreasing to 60.8 % in 2 years [6]. A recent report from Japan showed that the success rate was higher (83 % at 3 years) if trabeculectomy was preceded by a preoperative intravitreal injection of Bevacizumab [22]. A publication from Korea reported a success rate of 79 % at 1 year decreasing to less than 60 % at 2 years [23]. As our numbers were small we could not report the outcomes for each intervention separately and our results cannot be easily compared to previous reports. However our findings do reflect the reality of management, outcome and poor follow up even in a tertiary care center China. The high success rate at 1 year could be due to inclusion criteria, exclusion of patients with follow up less than 6 months as well as loss to follow up.

Cox proportional hazards model showed that factors like systemic disease, lens status, complications, anti-VEGF agents, PRP and prior-intraocular surgery did not influence the treatment outcome of NVG, but our numbers are small. While, anti-VEGF agents may be beneficial for the management of NVG reducing the need for glaucoma surgery and decreasing complications in eyes with neovascular glaucoma, the addition of intravitreal bevacizumab did not seem to increase the success rate in previous studies [24, 25]. All cases that required PRP underwent the procedure, but its influence on incidence of NVG is not clear [26, 27]. While PRP eliminates the stimulus for neovascularization it does not affect existing peripheral anterior synechiae (PAS) [28].

The complications encountered following intervention in this study were those usually reported after such interventions. Shallow anterior chamber was the most frequent early complication (8.5 %), while hypotony was the most frequent late complication (5.6 %). The extreme low complication rate could not compare with previous reports from Asian directly [6, 29]. It is very likely that the discrepancy between complication rates in our study and others are related to the poor follow up rates in the current study (i.e. patients with mild complications mostly like lost follow-up) and some data unavailable in this retrospective study. Given the limitations of sample size we too found that larger IOP reductions could be achieved with tube surgery compared to TSCPC but the postoperative complications were higher and included some sight threatening ones [30]. Although we did not encounter any endophthalmitis, phthisis and severe pain requiring enucleation occurred in 5 patients, three of those in the TSCPC group.

The limitations of the study include the retrospective design, exclusion of a large number of cases due to lack of follow up and lack of data as well as the lack of standardized management. The lack of a preferred practice or standardized regime also meant that the numbers for some interventions were too small to conduct any analysis. The fact that the management is affected by insurance status and personal finances made it difficult to create a protocol or standardize procedures, but this as well as poor follow up is the reality in China. This case series does however demonstrate the changing etiology of NVG in China, has the longest follow-up of NVG in an Asian population and reflects the reality of management in China.

## Conclusion

Diabetic retinopathy was the commonest detected cause of NVG encountered in a tertiary hospital in China. Cases present late, follow up is poor and management is partly determined by surgeon preference, insurance cover and personal finances. The increasing prevalence of diabetes in China will likely lead to a higher incidence of NVG and other related complications.
